# Towards the Development of a 3-D Biochip for the Detection of Hepatitis C Virus

**DOI:** 10.3390/s20092719

**Published:** 2020-05-10

**Authors:** Mariia Antipchik, Dmitry Polyakov, Ekaterina Sinitsyna, Apollinariia Dzhuzha, Mikhail Shavlovsky, Evgenia Korzhikova-Vlakh, Tatiana Tennikova

**Affiliations:** 1 Institute of Macromolecular Compounds, Russian Academy of Sciences, Bolshoy pr. 31, 199004 St. Petersburg, Russia; volokitinamariya@yandex.ru (M.A.); kat_sinitsyna@mail.ru (E.S.); 2 Institute of Experimental Medicine, Academician Pavlov’s str. 12, 197376 St. Petersburg, Russia; ravendoctor@mail.ru (D.P.); mmsch@rambler.ru (M.S.); 3 Institute of Chemistry, Saint-Petersburg State University, Universitesky pr. 26, Petrodvoretz, 198584 St. Petersburg, Russia; polinadzhuzha@mail.ru (A.D.); tennikova@mail.ru (T.T.)

**Keywords:** macroporous monolithic polymer layers, biochips, microarray, virus-mimetic particles, hepatitis C

## Abstract

The early diagnostics of hepatitis C virus (HCV) infections is currently one of the most highly demanded medical tasks. This study is devoted to the development of biochips (microarrays) that can be applied for the detection of HCV. The analytical platforms of suggested devices were based on macroporous poly(glycidyl methacrylate-*co*-di(ethylene glycol) dimethacrylate) monolithic material. The biochips were obtained by the covalent immobilization of specific probes spotted onto the surface of macroporous monolithic platforms. Using the developed biochips, different variants of bioassay were investigated. This study was carried out using hepatitis C virus-mimetic particles (VMPs) representing polymer nanoparticles with a size close to HCV and bearing surface virus antigen (E2 protein). At the first step, the main parameters of bioassay were optimized. Additionally, the dissociation constants were calculated for the pairs “ligand–receptor” and “antigen–antibody” formed at the surface of biochips. As a result of this study, the analysis of VMPs in model buffer solution and human blood plasma was carried out in a format of direct and “sandwich” approaches. It was found that bioassay efficacy appeared to be similar for both the model medium and real biological fluid. Finally, limit of detection (LOD), limit of quantification (LOQ), spot-to-spot and biochip-to-biochip reproducibility for the developed systems were evaluated.

## 1. Introduction

Hepatitis C virus (HCV) has been known as primary reason for chronic liver diseases representing a global social problem [1]. Most often, HCV is almost asymptomatic. The patients for decades do not even suspect that they are infected with the virus, and when the first symptoms begin to appear, the liver is already affected [2]. Thus, the development of effective, rapid, simple and accurate methods for early diagnostics of HCV is highly demanded. 

HCV is a small spherical pleomorphic RNA virus with buoyant density [3,4]. The diameter of HCV is in the range of 65–80 nm, while viral core ranges from 45 to 60 nm [5]. Three structural proteins of HCV are recognized: a core protein (p22), two envelope transmembrane glycoproteins (E1 and E2) and seven non-structural (NS) proteins that are involved in cleavage, assembly, transcription and some other functions. Some amount of the HCV can be associated with cellular lipoproteins, making so called “lipoviral particles”.

Currently, the diagnostics for HCV involves different serological tests [6,7,8]. The gold standard of serological analysis is the immunoassay-like enzyme, chemiluminescent, or fluorescent immunoassays [9,10]. However, these diagnostic methods have low efficiency at the early step of disease. It is explained by the fact that detectable level of antibodies against HCV cannot be reached earlier than 2–4 weeks after the manifestation of clinical symptoms at the acute step. At the asymptomatic step, the antibodies are produced in months. Moreover, they are not produced at all at the cyclic variant of HCV because of the low concentration of the virus particles in the blood. Finally, the detection of only HCV-specific immunoglobulins could not be counted as a confirmation of the active HCV infection due to the remaining anti-HCV antibodies in the organism for a longtime after viral clearance. Besides the immunoassays, a molecular test for HCV RNA is recommended to support the diagnosis and identify the specific HCV genotype [8]. Despite the robustness of molecular methods, one of their most important drawbacks is the complexity of required laboratory equipment. An alternative to immunoassay and molecular tests is a detection of HCV core or surface antigen(s), the level of which correlates with an infection state. HCV core/surface antigen(s) could be detected using molecular recognition principle and the same platforms as for anti-HCV antibodies [8]. 

One of the promising bioanalytical tools for rapid and highly sensitive detection of target components in biological fluids is biochip-based techniques [11,12]. To detect the virus infections, including hepatitis B and C, the development of protein [13,14,15,16] and DNA [17,18] biochips has been known. In the case of hepatitis analysis, the dominating biochip technique is represented by protein microarrays, where virus antigens are applied as probes whereas the detecting molecules are antibodies from a blood serum [19,20,21,22]. However, the antibody-bearing microarrays for the detection of virus antigens are also known [23].

It is obvious that the features of the solid support used for molecular recognition can influence the process of specific complex formation realized on a solid support. Usually, two-dimensional (modified glass) [24] or three-dimensional (hydrogel or nitrocellulose) matrices [12,25] are applied as platforms for biological microchips (microarrays). The important advantage of three-dimensional matrices is the higher probe loading and, as a result, an increase of bioassay sensitivity [26,27]. Moreover, the porous structure of 3-D matrices filled with aqueous media can provide for biomacromolecules a hydrophilic environment similar to that at solution. 

Regarding to hepatitis detection, 3-D sol-gel based microarrays [14,21] and microarrays based on commercially available nitrocellulose membranes [19,20], as well as 2-D microarrays representing gold-covered slides functionalized with poly(ethylene glycol) [22] and non-porous polymeric supports grafted with poly(glycidyl methacrylate) [23], have been elaborated. 

In this work, the macroporous monolithic material has been offered as a substrate for the construction of 3-D microarray for HCV detection. This kind of supports has a number of features differing them from other 3-D platforms described in current literature [27]. In contrast to hydrogels, the macroporous monoliths have a permanent porous structure, which is stable both in a dry state and in aqueous or aqueous-organic media due to a highly cross-linked polymeric network. Depending on the composition of polymerization mixture the pore size of such materials can vary from 100 nm to 2–3 µm [28]. Thus, the macroporous structure of the polymer matrix, filled with liquid, provides the in-solution-like conditions, prevents the biochip over-drying during the manipulations and favors the preservation of biomacromolecules’ biological activity. The widely used nitrocellulose membranes are also macroporous materials, which are usually characterized with a pore size in the range of 100–450 nm. However, this polymer does not have any own reactive groups suitable for the covalent immobilization of a probe. This obstacle can be overcome by modification of its surface with other polymers [29]. In turn, synthetic macroporous monolithic layers allow for variation not only in pore size but also in reactive functionality and hydrophilic/hydrophobic balance at in situ preparation step. At the same step, they can be covalently bound to a glass surface that provides high stability towards manipulations, e.g., washing under intensive shaking, drying, solvent changing, etc. 

Initially, the unique properties of macroporous monolithic materials have been found to be attractive for the development of biorecognition systems, in particular, sorbents for affinity chromatography [30] and flow-through solid-phase biocatalysis [31]. A decade ago, macroporous polymethacrylate monolithic matrixes were suggested as the supports for protein biochips [32,33]. The first polymer matrix that was developed as a platform for microarray and demonstrated promising bioanalytical results was macroporous monolithic material based on a copolymer of glycidyl methacrylate with ethylene dimethacrylate [P(GMA-*co*-EDMA)] [32]. Furthermore, the efficiency of macroporous monolithic platforms has been demonstrated also for DNA assay [34,35] and enzyme-reactor on a chip [36]. The influence of the matrix material and pore characteristics on the effectiveness of bioassay has been studied [36].

Taking into account the suitability of macroporous monoliths for the high-performance liquid chromatography (HPLC) of large objects such as viruses [37,38], that is the accessibility of sorbent porous space for viruses, in the present study, we focused on the development of biochip for the detection of hepatitis C virus. The study was carried out using hepatitis C virus-mimetic particles (HC VMPs), representing the polymer nanoparticles with the size close to HCV (70–80 nm) [5] and bearing surface virus antigen (E2 protein). VMPs mimic physico-chemical characteristics of the virus and, at the same time, they are cheaper and safer candidates for the development of analytical tools [39]. The suitability of VMPs as the models reproducing the behavior of real viruses has been verified in many works [39,40,41,42].

It is known that HCV penetrate inside the cells via receptor-mediated endocytosis [43]. The envelope protein E2 is one of the key virus antigens, which is responsible for the interaction with CD81 cell receptor [44]. It was established earlier that the E2 binding domain on CD81 was located in the large extracellular loop (CD81-LEL). To develop the biochip for HCV detection, we selected CD81-LEL or anti-E2 antibodies as the probes. At first step, the conditions for the specific binding of immobilized CD81-LEL/anti-E2 antibodies with E2 protein were optimized and then transferred to the procedure of HC VMPs’ detection. The possibility of analyzing the HC VMPs in spiked human blood plasma was demonstrated. 

## 2. Materials and Methods

### 2.1. Reagents and Supplements 

Glycidyl methacrylate (GMA, 97% pure), di(ethylene glycol) dimethacrylate (DEGDMA, 98% pure), 2-hydroxy-2-methylpropiophenone (Darocur-1173, 97% pure), 3-(trimethoxysilyl)propyl methacrylate, bovine serum albumin (BSA) and Cy3 dye were purchased from Sigma-Aldrich (Darmstadt, Germany). Hydrofluoric acid, dodecanol, cyclohexanol and ethanol were obtained from Vecton Ltd. (St. Petersburg, Russia). Recombinant CD81-LEL was purchased from ServiceGen (St. Petersburg, Russia). Polyclonal antibodies to E2 were products of GeneTeX (Hsinchu, Taiwan). All buffer solutions were prepared by dissolving the analytical-grade salts in deionized water and additionally purified by filtration through a 0.45-μm Milex Millipore microfilter (Merck, Darmstadt, Germany). The glass slides with dimensions of 25 × 75 × 1.2 mm were obtained from BioVitrum (St. Petersburg, Russia). 

### 2.2. Instrumentation 

The 125-W mercury lamp (Philips, Amsterdam, The Netherlands) of wide radiation spectrum and constant intensity was used for free-radical photo-initiated polymerization. The monolith morphology was studied using a scanning electron microscope, JSM-35 CF JEOL (JEOL, Tokyo, Japan). The mean pore size and pore size distribution were determined by automated standard porosimeter Porotech 3.1 (Porotech, Toronto, ON, Canada).

Nano Plotter NP 2.1 GeSiM (Radeberg, Germany) was used for non-contacting spotting ligands for the immobilization on a microarray polymer surface. The procedure of biochip washing was carried out using orbital shaker Unimax 1010 Heidolph (Schwabach, Germany). The special secure seal hybridization chambers Grace Biolabs (Bend, OR, USA) were used to perform the coupling of immobilized ligands with analytes. The microarrays were scanned using Scanner GenePix 4000 B, Axon Instruments (Foster, CA, USA). GenePix 6.0 software was used to analyze the data.

Thermomixer Comfort Eppendorf (Hamburg, Germany) was applied for the reaction of antibody labeling with fluorescent dye Cy3. The removal of unbound dyes was performed by ultrafiltration using centrifuge Sigma 2-16 KL (Taufkirchen, Germany) and Sartorius Vivaspin membrane with MWCO 30,000 (Stedium, Germany).

### 2.3. Synthesis and Characterization of Monolithic Layers

The well of 20 × 40 × 0.2 mm was prepared via the acidic etching of a glass surface with 30% solution of hydrofluoric acid. The prepared wells were washed with water until neutral pH, then with 0.1 M sodium hydroxide and, finally, with water again until neutral pH. The surface of fabricated wells was silanized using a 20% solution of 3-(trimethoxysilyl)propyl methacrylate in toluene for 20 h in a dark at room temperature. After the reaction complete, the glasses were washed three times with acetone and then with ethanol, and dried.

Macroporous monolithic layers were synthesized using UV-initiated free-radical copolymerization of GMA and DEGDMA inside the fabricated glass wells. In total, 3 mL of polymerization mixture for the production of 15 layers consisted of 0.78 mL DEGDMA and 0.42 mL GMA (monomers), 0.9 mL dodecanol and 0.9 mL cyclohexanol (porogens), as well as 0.013 g 2-hydroxy-2-methylpropiophenon (initiator, 1% from the mass of monomers). Before irradiation, the polymerization mixture was preliminarily purged with dry nitrogen for 5 min and then 200 μL of polymerization mixture was added per well. The polymerization was initiated using UV-irradiation at room temperature for 20 min. After the polymerization process was completed, all polymer layers were washed with ethanol, a mixture of ethanol with water (1/1) and pure water for 3 h, changing solvent portions every 30 min, and were dried at 40 °C. The porous characteristics and morphology of the surface of copolymers were investigated using etalon porosimetry and scanning electron microscopy.

### 2.4. Biochip Printing 

To create the biochip for the HCV detection, two types of probes were used for immobilization on the surface of macroporous monolithic layers, namely, recombinant CD81-LEL and polyclonal antibodies against the E2 protein of HCV. To establish optimal probe concentration, probe solutions in 0.01 M sodium phosphate buffer (PBS, pH 7.4) with different concentrations were spotted onto the surface (100 replicates for each concentration). The spotted sample volume was equal to 200 pL. Probe concentration varied from 0.2 to 2.5 mg/mL for CD81-LEL and from 0.2 to 4.0 mg/mL for anti-E2 antibodies. The slides placed in the special chamber were incubated for 24 h at 37 °C for probe covalent immobilization. After that, the slides were consequently washed with 0.01 M PBS, 2 M NaCl and 0.01 M PBS; the time of washing procedure was 10 min for each solution. Then, the slide’s surface was blocked with 1% BSA in PBS for 45 min at slight stirring, and ready-to-use biochips were finally washed with three portions of PBS for 15 min. 

### 2.5. E2 Production

A plasmid containing the E2 gene fragment and the superfolder green fluorescent protein (GFP) gene has been created. The E2 core sequence (412-645 aa) [43] was obtained by PCR using forward and reverse primers, respectively: 5′-ctggaattcCAGCTGATCAACACC-3′ and 5′-ctgagctcAGACTGGAAGTACAGGTTTTCGTTGCAAGCAGCTTC-3′. The HCV cDNA genotype 1a, isolate 1, was used as a template for PCR. The PCR product obtained was subcloned into the plasmid for the synthesis of chimeric E2-superfolder GFP [45,46]. The nucleotide sequencing showed that the final plasmid comprised the E2 core gene and the superfolder GFP gene in the same reading frame. The chimeric protein E2-superfolder GFP synthesized in *Escherichia coli* (*E. coli*) comprised the C-terminal sequence of 6-histidines for affinity purification. The target protein was synthesized in *E. coli* BL21 (DE3) cells and isolated on Ni-agarose. The synthesis of recombinant chimeric protein was induced by the addition of isopropyl β-d-1-thiogalactopyranoside IPTG (0.25 mM) at OD600 0.6 for 16 h at 37 °C. After this, the cells were centrifuged, washed with PBS, sonicated and centrifuged again to separate soluble and insoluble cell fractions. The target E2-GFP was found both in the soluble cell fraction (in small amounts: not more than 100 μg per liter of culture medium) and in inclusion bodies (3–10 mg per liter of culture medium). The insoluble cell fraction containing inclusion bodies was dissolved in 8 M urea and purified on an Ni-agarose affinity column. The resulting E2-GFP protein was loaded on SDS-PAAG electrophoresis. The gel was stained with Coomassie R250. The major band between 45,000 and 66,000 corresponding to the molecular mass of the target protein E2-superfolder GFP (54,000) was cut from the gel and subjected to trypsinolysis. Mass spectrometry analysis of tryptic fragments showed the presence of the peptides of the E2 sequence.

### 2.6. Labeling of Antibodies

Fluorescently labeled anti-E2 antibodies were obtained using Cy3 fluorescent dye. Initially, stock solution of Cy3 in DMSO with a concentration of 1.0 mg/mL was prepared. The molar ratio of antibody:Cy3 was 1:5. The reaction proceeded for 30 min at 25 °C in 0.01 M PBS, pH 7.4, at stirring. The removal of unbound dye was performed by ultrafiltration using Vivaspin membrane with MWCO 30,000 at 7000 g and a temperature of 5 °C for 15 min. 

### 2.7. Preparation HC VMPs

#### 2.7.1. Preparation of Nanoparticles 

For the preparation of HC VMPs, we used the nanoparticles based on block-copolymer consisting of poly(D,L-lactic acid) (PLA) and poly(ethylene glycol)-5000 methyl ether (PLA-*b*-PEG). The copolymer was synthesized by the ring opening polymerization of D,L-lactide with the use tin(II) octoate as catalyst and PEG-5000 methyl ether as initiator (PLA/PEG = 1000; PLA/tin(II) octoate = 600). The polymerization was performed in bulk for 4 h at 135 °C. The polymer was dissolved in CHCl3 and precipitated into methanol. The precipitate was dried in a vacuum until the constant weight. The molecular weight characteristics of PLA-*b*-PEG was determined by size-exclusion chromatography in tetrahydrofuran at 40 °C using a Shimadzu LC-20 Prominence system with a RID 10-A refractometric detector (Kyoto, Japan) and polystyrene standards. The number average (*M_n_*) and weight average (*M_w_*) molecular weights were found as 96,000 and 133,000, respectively, and the dispersity (*Đ*) was 1.4.

The nanoparticles were prepared using the nanoprecipitation technique: a solution of PLA-*b*-PEG in acetonitrile (solvent) with the concentration of 0.5 mg/mL was slowly dropped under stirring in water as a precipitant. The stirring speed of the system was 1000 rpm and the rate of introduction of organic phase into the aqueous phase was 2 mL/min. The yield of polymer particles corresponded to 93%. The ratio of organic phase to water was 1:5. The average hydrodynamic particle size (*D_H_*) and polydispersity index (PDI) were determined by dynamic light scattering (Zetasizer Nano ZS, Malvern Instruments, Worcester, UK). 

#### 2.7.2. E2 Immobilization 

To covalently modify the surface of nanoparticles with E2-GFP (E2), initially, the surface was activated by the treatment of nanoparticles with 0.01 M NaOH for 15 min at 23 °C, and the product was purified by dialysis against water for 1.5–2 h. Generated carboxylic groups were activated with hydroxysuccinimide (NHS) and N-(3-Dimethylaminopropyl)-N′-ethylcarbodiimide (EDC) in 0.01 M MES buffer (pH 5.6). In total, 0.8 mg of NHS (1 mg/mL) and 0.5 mg of EDC (1 mg/mL) were taken for 16 mg of nanoparticles. After 40 min of incubation at 4 °C, the nanoparticles were transferred into 0.01 M sodium borate buffer (pH 8.4) and 2 to 70 µg of E2 was added to the activated nanoparticle’s suspension. Non-bound protein was removed with the use of ultrafiltration trough the membranes with MWCO 100,000. The amount of unbound protein was determined spectrophotometrically at 490 nm. The amount of immobilized protein was calculated as a difference between initial and unbound protein amounts (Equation (1)):

*Q_imm_ = Q_initial_ − Q_unbound_* (1)

where *Q_imm_* is the mass of immobilized protein (µg); *Q_initial_* is the initial mass of protein taken for immobilization (µg); *Q_unbound_* is the mass of unbound protein after immobilization (µg).

The nanoparticles bearing E2 were transferred into 0.01 PBS (pH 7.4) and stored at 4 °C. The E2 loading ranged from 2 to 70 μg per mg of nanoparticles. Additionally, after modification, VMPs were monitored by nanoparticle tracking analysis (NTA) with the use of Nanosight NS300, Malvern (Worcester, UK).

### 2.8. Bioanalisys 

#### 2.8.1. Direct Analysis

A 500-µL solution of E2 or HC VMPs with a protein concentration ranging from 0.1 to 2.5 µg/mL in 0.01 M PBS, pH 7.4, was introduced into special hybridization cells for affinity binding, which was fixed to the supporting glass of biochip. The slides were incubated in the dark for 1–3 h at 37 °C and 300 rpm on orbital shaker. After coupling, the slides were washed using the following washing buffers: 2 × SSC for 5 min, 1 × SSC for 5 min and 0.5 × SSC for 5 min. Finally, the microarrays were dried with air flow generated by a compressor and then scanned at a wavelength of 530 nm. 

#### 2.8.2. Sandwich-Analysis 

The affinity binding of E2 and HC VMPs to biochip was the same as described above. After affinity coupling, the slides were washed with 0.01 M PBS, pH 7.4, for 15 min, and then 500-µL solution of anti-E2 antibodies labeled with Cy3 with a concentration ranging from 0.1 to 2.5 µg/mL in 0.01 M PBS, pH 7.4, was introduced into a hybridization cells for affinity binding. The affinity binding with antibodies was carried out in the dark for 2 or 3 h at 37 °C and 300 rpm on an orbital shaker. After coupling, the slides were washed using the following washing buffers: 0.5 × SDS in 2 × SSC for 5 min, 1 × SSC for 5 min and 0.5 × SSC for 5 min. Finally, the microarrays were dried with flow of air supplied by a compressor and then scanned at a wavelength of 530 nm. 

### 2.9. Statistics and Reproducibility, LOD and LOQ 

The data of analysis were expressed as mean ± SD (*n* = 100). To analyze the statistical significance among the groups, one-way analysis of variants (ANOVA) in Excel with the XLSTAT was used. *p* ≤ 0.05 was counted as a statistically significant. 

The reproducibility of analytical results was estimated with the use of variation factor (K):

*K = σ / Ī × 100%* (2)

where *K* is the variation factor (%), σ is the standard deviation of the fluorescence intensity, *Ī* is the mean fluorescence intensity. *K_1_* characterized the spot-to-spot reproducibility (*n* = 100) inside a biochip and *K_2_* reflected biochip-to-biochip reproducibility (*n* = 5). 

The limit of detection (LOD) and limit of quantification (LOQ) were calculated from the data of linear regression of the calibration plot built for concentrations from 50 to 500 ng/mL according to Equations (3) and (4):

*LOD = 3 × S_y/x_ / b* (3)

*LOQ = 10 × S_y/x_ / b* (4)

where *S*_*y*/*x*_ is the standard deviation of the regression residuals and *b* is the slope of the regression line [47]. The plots were built in coordinates’ “signal-to-noise ratio (SNR)—E2 concentration (ng/mL)”. The signal-to-noise ratio (SNR) was calculated as:

*SNR = (SM − BM) / SD* (5)

where *SM* is the signal mean, *BM* is the background mean, *SD* is the standard deviation of the background.

## 3. Results and Discussion

### 3.1. Preparation of Biochips 

It is well known that the solid support can significantly influence the processes occurring on its surface including the ones based on a principle of molecular recognition. Recently, we studied the effect of average pore size of macroporous biochip’s platform on the relative signal intensity [36]. It was established that the materials with pore size of 300–400 nm provided the achievement of a plateau for relative signal intensity significantly faster than those with a pore size larger 1 μm. Moreover, the more hydrophilic materials showed the higher relative signal intensity and, as a result, the lower limit of detection [36]. In particular, the best analytical potential in the analysis of proteins was found for the material based on the copolymer of glycidyl methacrylate (GMA) and di(ethylene glycol) dimethacrylate (DEGDMA), where GMA and DEGDMA were a functional monomer and a hydrophilic cross-linker, respectively. 

**Figure 1:**
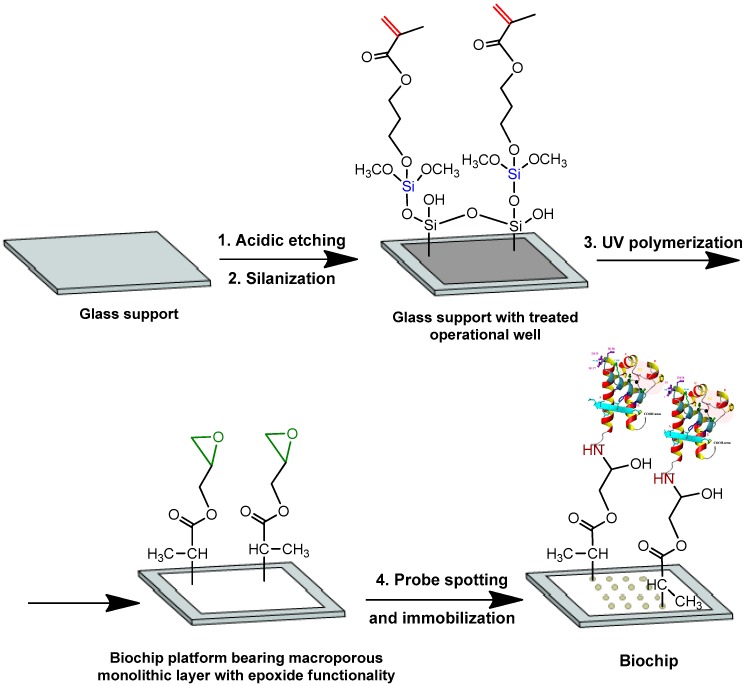
Scheme of biochip preparation: (1) glass etching with hydrofluoric acid; (2) silanization with 3-(trimethoxysilyl)propyl methacrylate; (3) UV polymerization of glycidyl methacrylate (GMA) and di(ethylene glycol) dimethacrylate (DEGDMA); and (4) probe spotting and immobilization via the reaction between the epoxy groups of the polymer layer and the amino groups of the protein.

Based on that research, in this work, we selected P(GMA-*co*-DEGDMA) to prepare 3-D biochip platforms. Since the macroporous polymer layers are thin and fragile, they were covalently attached to the glass supporting slide to simplify the manipulation. The scheme of biochip platform preparation includes several steps: (1) the preparation of operative wells by acidic etching and further reaction with methacrylate-bearing silane to provide the covalent attachment of polymer layer; (2) the polymerization of the polymer layer inside the well, and (3) probe spotting and its covalent immobilization (Figure 1). 

The synthesis of P(GMA-*co*-DEGDMA) was carried out by free radical photoinitiated polymerization of GMA and DEGDMA in the presence of dodecanol and cyclohexanol as porogenic solvents and 2-hydroxy-2-methylpropiophenone as initiator. According to the data of etalon porosimetry (Figure S1), all developed materials were characterized by narrow pore size distribution and had the following characteristics: average pore size = 360 ± 20 nm; porosity = 60 ± 2%; and specific surface area = 10.2 ± 0.3 m^2^/g. The surface morphology was evaluated by scanning electron microscopy (SEM) (Figure S2). The main feature of methacrylate-based monoliths is the total similarity of porous structure both in the volume and on the surface. The substrates obtained were characterized with a typical, for such kind of materials, microglobular structure pierced with interconnected macrochannels [28]. The cross-linked polymer microglobules were uniformed, indicating a high structure homogeneity. 

Since P(GMA-*co*-DEGDMA) possesses with the epoxy groups, which are highly reactive towards amino-bearing compounds, the covalent immobilization of a protein probe can be realized. In our case, the probes (in 0.01 M PBS, pH 7.4), namely CD81-LEL or anti-E2 antibodies, were covalently immobilized in spots on the surface of macroporous polymer supports for 20 h at 37 °C (Figure 1). After immobilization, the polymer surface was blocked with BSA to reduce the surface noise of polymer material, which can distort the results of analysis.

### 3.2. Preparation of HC VMPs

It is known that HCV is a small RNA virus with a diameter of approximately 70–80 nm, where 50–60 nm is a size of virion [5]. As it was mentioned above, one of the main envelope proteins of HCV is E2 protein, which is responsible for virus entering inside the cells. Thus, to prepare the physico-chemical model of HCV, we used nanoparticles (NPs) of close size and modified their surface with recombinant E2 protein. The mimicking nanoparticles were papered from poly(D,L-lactic acid)-*b*-poly(ethylene glycol) (PLA-*b*-PEG) and possessed with the following characteristics: average hydrodynamic diameter (*D_H_*) and polydispersity index (*PDI*) were 60 ± 5 nm and 0.07, respectively (Figure S3). 

**Figure 2:**
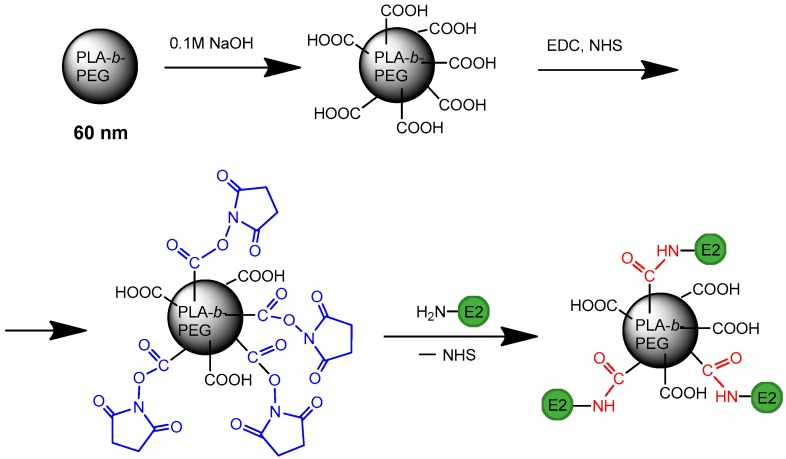
Scheme of the preparation of hepatitis C virus-mimetic particles (HC VMPs).

To modify PLA-*b*-PEG nanoparticles, initially, their surface was slightly hydrolyzed with 0.01 M sodium hydroxide solution to generate carboxylic groups (Figure 2). The activation of generated carboxyls was carried with the use of water-soluble carbodiimide (EDC) and N-hydroxysuccinimide (NHS) to obtain NHS-activated esters that were highly reactive towards amino-bearing (macro)molecules. To find the most optimal E2 loading, which would provide the efficient VMPs binding to the probe immobilized on the biochip surface, as well as the appropriate detection signal, we prepared the set of modified NPs with 2, 5, 15, 30, 43 and 59 µg of protein/mg NPs. In the range of protein loading from 2 to 15 µg per mg of NPs, the VMPs had the same *D_H_* and *PDI* as initial NPs. For VMPs with E2 loading equal to 30 µg/mg of NPs, *D_H_* was slightly increased to 75 ± 8 nm (PDI = 0.23) but it was still appropriate to be the virus model. The increase of target protein loading up to 43 µg/mg NPs was followed by the increase in the average diameter of NP_S_ (132 ± 47) and remarkable PDI broadening (0.65). A further increase in E2 loading provided the formation of bimodal size distribution that was inappropriate for the consideration of them as VMPs. Thus, the VMPs containing from 2 to 43 µg of E2 per mg of NPs were selected for the development of bioanalysis on a chip. 

**Figure 3:**
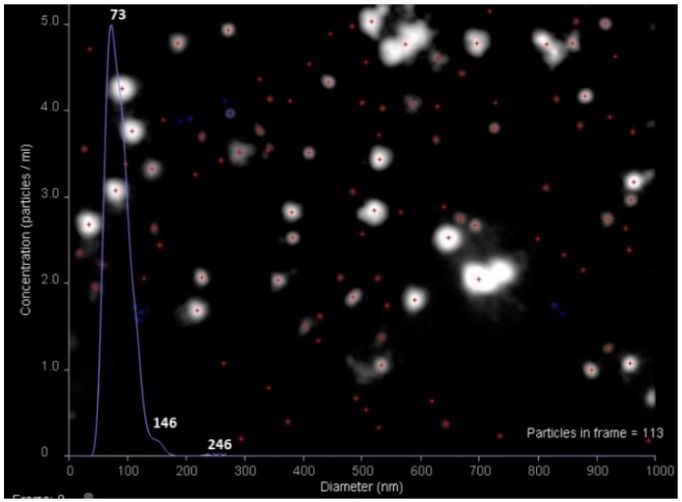
Nanoparticle tracking analysis (NTA) of VMPs containing 30 µg of E2/mg nanoparticles (NPs).

Additionally, the size of VMPs containing 30 µg of protein/mg NPs was evaluated by nanoparticle tracking analysis (NTA) and scanning electron microscopy (SEM). As seen from Figure 3, the main fraction of VMPs had the diameter equal to 73 ± 11 nm, which was in a good agreement with the measurements by dynamic light scattering (DLS). The analysis of the same VMPs by SEM was complicated by strong heating and partial polymer melting under an electron beam during investigation (Figure S4) that led to some loss of sharpness of borders and the distortion of the spherical shape of nanoparticles. In spite of that, the image analysis discovered the average diameter of nanoparticles 95 ± 25 nm that was close to the data of DLS and NTA. 

### 3.3. Analysis on Biochips

**Figure 4:**
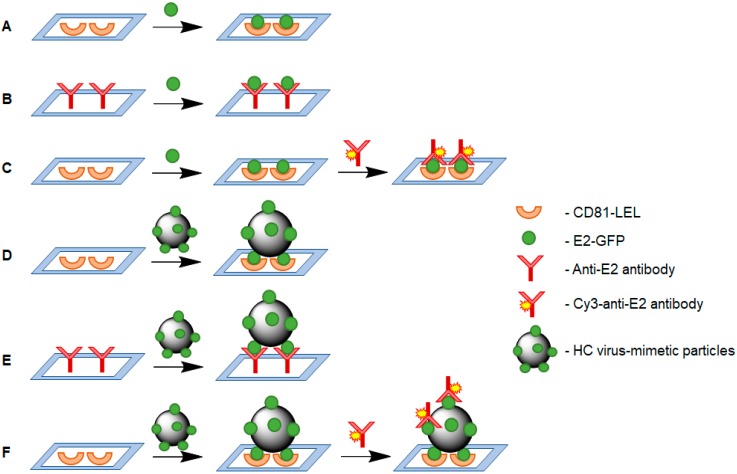
Schemes of different variants of affinity interactions: (**A**–**C**)—the interaction of probes with E2 protein; (**D**–**F**)—the interaction of probes with HC VMPs; (**A**,**B**,**D**,**E**)—the direct analysis; (**C**,**F**)—“sandwich” analysis.

The development of efficient analytical procedure included several steps, such as the study of the effects of probe loading, affinity binding time, analyte concentration in a solution, etc., on detection signal. For optimization, we used the direct mode of analysis, when the detection was performed directly via the measurement of the fluorescence of GFP conjugated with E2. Finally, the optimized analytical protocol was transferred to the sandwich-analysis allowing for HCV detection in real biological samples. The considered analytical schemes are summarized in Figure 4. In order to analyze the results, the values of relative signal intensity, namely the difference between the mean signal intensity detected in the spots and the mean background intensity, were used for comparison. In contrast to the direct mode, in the “sandwich” method, the affinity complexation was detected by measuring the fluorescence of Cy3 used for the labeling of covering antibodies. 

### 3.4. Optimization of Analytical Procedure 

#### 3.4.1. Probe Amount 

**Figure 5:**
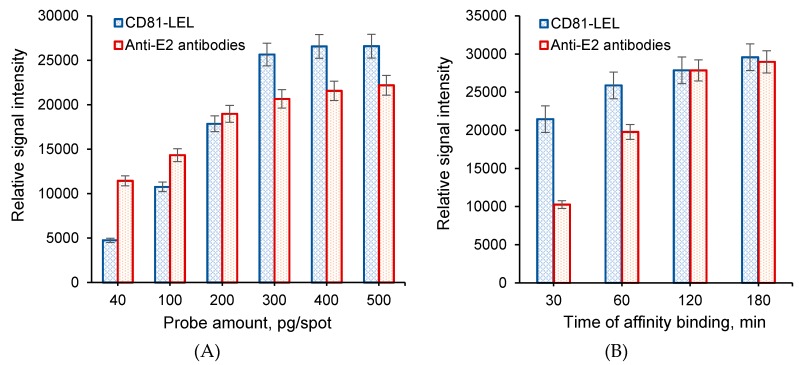
Effect of probe amount on relative signal intensity (**A**) and interaction time (**B**). Conditions: temperature—37 °C; E2 concentration was 500 ng/mL; (**A**) interaction time—2 h; (**B**) probe amount—300 pg/spot.

At this step, the affinity interactions of CD81-LEL or anti-E2 antibodies with E2-GFP (E2) as an analyte were studied (Figure 4A,B). To establish the most optimal probe amount, the solutions of CD81-LEL or anti-E2 antibodies of different concentrations were spotted at fixed volume (200 pL) onto the surface of macroporous polymer supports. The probe concentrations were varied in the range from 200 to 2500 μg/mL that corresponds to 40–500 pg of probe per spot. The analysis was carried using an E2 solution with a concentration of 500 ng/mL. For both types of probes, the increase in probe amount on the surface was accompanied with the gradual increase in relative signal intensity until 300 pg of probe/spot (Figure 5A). Over this probe amount, no drastic increase in relative signal intensity was observed. Therefore, the minimal probe amount providing a high analytical signal was found to be 300 pg/spot. In addition, the comparison of CD81-LEL and anti-E2 antibodies as immobilized probes indicated the higher binding of analyte to CD81-LEL than that detected for polyclonal antibodies. 

#### 3.4.2. Interaction Time 

The optimal time of interaction was chosen for the both types of affinity pairs, namely antigen–antibody (E2–anti-E2 antibodies) and ligand–receptor (E2–CD81-LEL). The time of affinity binding was varied from 30 min to 3 h. It was found that the interaction of E2 with CD81-LEL for 30 min gave the appearance of low detectable signal (Figure 5B). The increase in interaction time to 1 and 2 h caused a growth in relative signal intensity, but the further extension in reaction time to 3 h did not lead to a significant change in fluorescence intensity. A similar tendency was observed for the interaction of E2 with antibodies. Thus, the time of 2 h was chosen for further experiments.

#### 3.4.3. Analyte Concentration 

**Figure 6:**
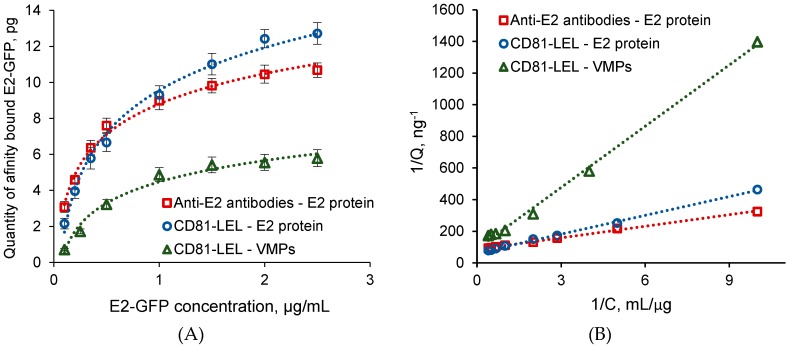
Adsorption isotherms (**A**) and their linearized forms (**B**) built for E2 binding to two kinds of biochips. Equations for linearized isotherms (B) obtained for E2 binding to: CD81-LEL: y = 39.589x + 63.467 (R^2^ = 0.9984); anti-E2 antibodies: y = 24.304x + 85.94 (R^2^ = 0.9967); and for VMPs binding to CD81-LEL: y = 129x + 90.542 (R^2^ = 0.9964). Conditions: temperature—37 °C, time of affinity binding—2 h, probe amount—300 pg/spot.

A series of analysis with the use of two kinds of biochips was carried out. The concentration of analyte in both cases was varied from 0.01 to 2.5 µg/mL. The adsorption isotherms were built in coordinates’ “amount of bound analyte–concentration of analyte solution”. The amounts of bound analyte were calculated from the data on relative signal intensity using those preliminary plotted for the E2 calibration curve. According to the data shown in Figure 6, the maximum amount of E2 bound to the probe immobilized on polymer surface was reached in both cases at analyte concentration equal to 2.0 µg/mL. The results also indicate that the biochips bearing CD81-LEL as a probe demonstrate a greater capacity of analyte binding in comparison to the case of antibodies. 

In our previous study, we demonstrated the applicability of macroporous polymer biochips for the determination of the apparent dissociation constants (*K_diss_*) and established the independence of these values on the probe concentration and pore size of macroporous monolithic supports [36]. Thus, it was demonstrated that the developed supports do not affect the process of biomolecular recognition and allow for the study of the affinity complex formation in the uncomplicated form.

To estimate the quantitative parameters of affinity binding for two kinds of pairs, e.g., antigen–antibody (E2–anti-E2 antibodies) and ligand–receptor (E2–CD81-LEL), the plotted adsorption isotherms (Figure 6A) were linearized in double reciprocal coordinates (Figure 6B). The calculated values of apparent dissociation constants were found to be 5.2 ± 0.3 nM (R^2^ = 0.9967) for antigen–antibody interaction and to 11.6 ± 0.5 nM (R^2^ = 0.9984) for ligand–receptor binding.

### 3.5. Selection of VMPs and Comparison of Their Detection to Free Protein

**Figure 7:**
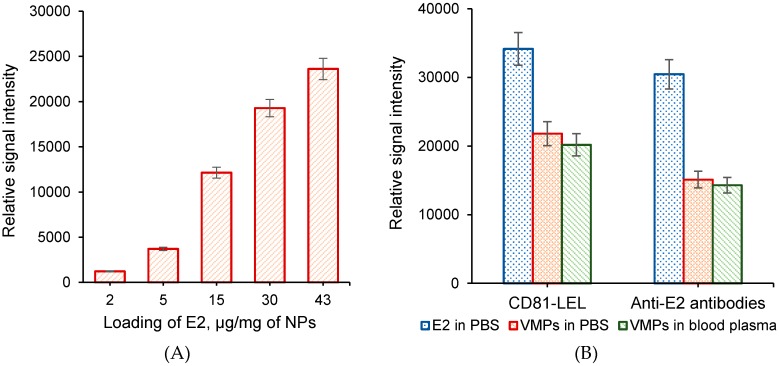
Comparison of relative signal intensity registered for different E2 loadings per mg of nanoparticles (**A**) and for the detection of free protein and VMPs in buffer solution and spiked human blood plasma (**B**). Conditions: probe amount—300 pg/spot, analyte concentration—1000 ng/mL, time of affinity binding—2 h, temperature—37 °C; (**A**) probe—anti-E2 antibodies, 0.01 M PBS (pH 7.4); (**B**) VMPs contained 30 µg/mg of NP_S_, 0.01 M PBS (pH 7.4) or human blood plasma.

The influence of E2 loading onto the surface of polymer nanoparticles on the efficiency of bioanalysis was investigated (Figure 7A). As it was described above, a set of VMPs bearing 2, 5, 15, 30 and 43 µg of E2 per mg of nanoparticles was prepared for the study. Only subtle signal intensity was detected for the suspension of VMPs containing 2 µg of E2/mg NPs. As expected, an increase in E2 content on the surface of nanoparticles led to the considerable growth of the relative signal intensity. Considering the broad polydispersity index for the sample containing 43 µg of E2/mg NPs, the most optimal loading of E2 was counted as 30 µg of E2/mg NPs. 

The affinity binding of selected VMPs to the biochips bearing CD81-LEL or anti-E2 antibodies as probes was carried out in 0.01 M PBS, pH 7.4, and spiked human blood plasma (Figure 7B). Additionally, the results obtained were compared to those observed for free protein affinity binding. A 40% and 50% decrease in relative signal intensity was observed for the binding of VMPs to CD81-LEL and anti-E2 antibodies, respectively, comparing free E2 binding to the same probes. This result can be explained by the much larger size of VMPs. Interacting with the probe, VMPs block larger than free protein surface area. As a result, less units of analyte can be bound to the surface area of one spot. 

The comparison of VMPs binding from buffer solution and spiked human blood plasma allows for the conclusion on similar affinity binding process. For both affinity pairs, namely, ligand–receptor and antigen–antibody, the relative signal intensities were the same. It means that the non-specific interactions, which are possible in a such complex biological fluid as a blood plasma, are neglectable. 

Figure 5A illustrates the dependence of binding efficiency (regarding to E2) on the concentration of VMPs. As seen, the maximal signal intensity was lower than for the same concentration of free protein. The probable reason for such a difference was discussed above. The minimal detected concentration was 50 ng/mL. The value of dissociation constant calculated from the linearized form of adsorption isotherm (Figure 6B) was found as 26.3 ± 0.7 nM (R^2^ = 0.9964) that was approximately twice as high as that established for free E2.

### 3.6. “Sandwich” Analysis

**Figure 8:**
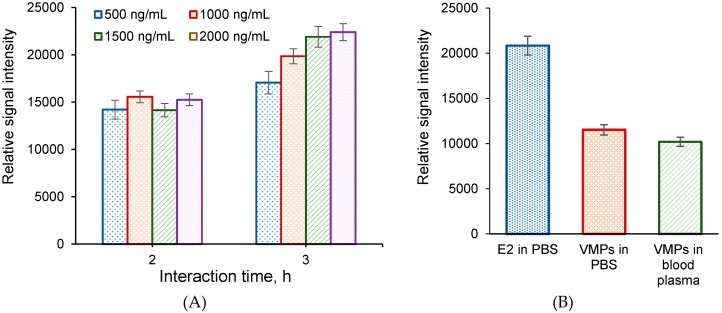
Comparison of “sandwich” analysis results for the detection of free protein at the conditions of variation of the affinity binding time and Cy3-labled antibodies’ concentration (**A**) and for the detection of VMPs in buffer solution and spiked human blood plasma (**B**). Conditions: probe—CD81-LEL, probe amount—300 pg/spot; analyte concentration—1500 ng/mL, temperature—37 °C; VMPs contained 30 µg/mg of NPS; the time of affinity binding for step 1 was 2 h; (B) the concentration of Cy3-labled anti-E2 antibodies was 1000 ng/mL; the time of affinity binding for step 2 was 3 h; 0.01 M PBS, pH 7.4, or human blood plasma were used as media.

To detect target components at real conditions, so-called “sandwich” method of analysis is usually applied. In this case, the analytical process is based on the affinity binding of E2 to CD81-LEL immobilized on the macroporous polymer support and subsequent interaction of bound E2 protein with Cy3-labeled anti-E2 antibodies (Figure 4D,F). CD81-LEL was used as a probe due to its better binding of E2 that was demonstrated in above described experiments. Based on the experiments on direct analysis, the following conditions for the first step of bioassay have been chosen as optimum: 300 pg/spot for CD81-LEL, 1.5 μg/mL for analyte concentration and 2 h for affinity binding. In order to establish the optimal conditions for the second affinity step, e.g., the binding of the Cy3-labeled anti-E2 antibodies to E2 or HC VMPs previously captured by biochip, the influence of important parameters such as affinity binding time for step 2 and the concentration of antibodies on the relative signal intensity have been investigated. The results presented in Figure 8A clearly indicate that the time of affinity binding plays a crucial role. In particular, the increase in binding time from 2 to 3 h led to the growth of relative signal intensity by 35%. Most probably, the low diffusivity of antibodies and steric limitations at their interaction with large target (VMPs) already bound to the probe located at rigid support slow down the rate of the secondary affinity process. In turn, the variation in antibodies’ concentration in the range from 500 to 2000 ng/mL did not provide any drastic improvement in binding efficiency. Thus, 3 h for affinity binding and 1000 ng/mL for concentration of antibodies were counted as optimal parameters for the realization of the second step of the “sandwich” analysis. 

The conditions optimized for free E2 were transferred to the detection of VMPs in both model buffer solution (0.01 M PBS, pH 7.4) and spiked human blood plasma (Figure 8B). As for the direct detection of VMPs, the detection efficacy of VMPs with the use of the “sandwich” technique was the same for such simple medium as buffer solution and the complex biological fluid as a blood plasma. It means that all target affinity interactions are very strong and non-specific interactions do not take place. The comparison of relative signal intensity registered for free E2 and VMPs after the “sandwich” analysis indicates the decrease in this parameter for the detection of VMPs by 45%–48%. The result obtained is expectable since the larger size of analyte can block larger surface area after the interaction with immobilized probe in a spot. Despite that, the data confirm the applicability of developed biochip for the detection of HC VMPs at the real conditions. 

### 3.7. LOD, LOQ and Reproducibility of Analysis

The performance of biochips at low analyte concentration (50–500 ng/mL) was analyzed to determine analytical detection limits, such as the limit of detection (LOD) and limit of quantification (LOQ). The summary of analysis is presented in Table 1. LOD and LOQ determined for free protein were higher than these calculated for the VMPs’ detection. However, it is worth noting that LOD and LOQ determined for analysis of VMPs were close for both the direct and “sandwich” variants of analysis. Moreover, LOD and LOQ calculated for the “sandwich” analysis performed in 0.01 M PBS (pH 7.4) were the same as for analysis in spiked human blood plasma. This fact indicates the high affinity of the selected probe to the object of interest and an absence of side none-specific processes interfering with the target interactions. 

The biochips under study were characterized by a high quality of spots and their uniformity as well as reproducibly (Figure S5). The coefficients of variation (*K*) were determined to evaluate the reproducibility of bioassays with the use of biochips obtained. *K* is calculated as the ratio of the standard deviation of fluorescence intensity to the mean fluorescence intensity. The intrafield coefficient of variation (*K_1_*) reflects the reproducibility of analytical signal from spot-to-spot inside the same biochip, whereas the interfiled coefficient of variation (*K_2_*) indicates the reproducibility of a signal from biochip-to-biochip. In the case of developed devices, *K_1_* and *K_2_* were calculated to be 5% and 17%, respectively, for the detection of VMPs using direct analysis and 9% and 18%, respectively, for the application of the “sandwich” technique. These results can be counted as reproducible and comparable or surpassed some commercial platforms [27,34,48,49,50]. Moreover, the standardizing of the procedure of polymer support fabrication can also favored to the improvement of analysis reproducibility.

## 4. Conclusions

Using the developed biochips, different variants of the bioassays were tested to detect E2 protein and hepatitis C virus-mimetic particles, including direct binding to immobilized CD81-LEL or anti-E2 antibodies as well as the “sandwich” approach. The optimal operating conditions for developed biochips were chosen by varying the probe amount, time of affinity binding, analyte concentration, etc. It was established that the affinity of E2 to anti-E2 antibodies (antigen to antibody) was higher than E2 to CD81-LEL (ligand to receptor). In turn, the application of CD81-LEL as a probe seemed to be preferable due to the higher amount of analyte, which could be bound from the medium. The principal applicability of the developed biochips and high reproducibility of the detection of large biological objects, such as VMPs, were demonstrated. The identical efficacy for VMPs’ detection in model buffer medium and spiked human blood plasma proved the suitability of biochips for performance of analysis in real biological fluids. Thus, both developed biochips and optimized analytical procedures can serve as a base for further studies towards practical application for hepatitis C virus detection.

## Figures and Tables

**Table 1:** Limits of detection (LOQ) and limits of quantification (LOQ) calculated for the different variants of analysis.

Probe	Analyte	Analysis	*S_x/y_*	*b*	*R^2^*	LOD, ng/mL	LOQ, ng/mL
Anti-E2 antibodies	E2-GFP	direct, in PBS	1.95	0.047	0.99151	124	414
CD81-LEL	E2-GFP	direct, in PBS	3.87	0.050	0.98482	232	774
Anti-E2 antibodies	VMPs	direct, in PBS	0.93	0.037	0.99361	75	251
CD81-LEL	VMPs	direct, in PBS	1.38	0.038	0.99093	108	363
CD81-LEL	VMPs	sandwich, in PBS	0.63	0.027	0.99248	70	233
CD81-LEL	VMPs	sandwich, in human blood plasma	0.69	0.028	0.99142	73	246

## Supplementary Materials

The following are available online at https://www.mdpi.com/1424-8220/20/9/2719/s1, Figure S1: Integral (A) and differential (B) distribution of pore volume (V) versus pore radius (log r), Figure S2: SEM image of macroporous monolithic material based on P(GMA-*co*-DEGDMA) (carbon deposition), Figure S3: Dynamic light scattering of PLA-*b*-PEG nanoparticles used for VMPs’ preparation, Figure S4: SEM image of PLA-*b*-PEG-based VMPs (gold deposition), Figure S5: Images of scanned biochips (fragments): (A) CD81-LEL (probe)–E2 (analyte); (B) CD81-LEL (probe)–VMPs (analyte).
