# Supplementary material for: Towards the Development of a 3-D Biochip for the Detection of Hepatitis C Virus
Source: Sensors (Basel). 2020 May 10;20(9):2719. doi: 10.3390/s20092719 (PMC7249126; doi:10.3390/s20092719)
Supplement: Supplementary file 1 [file sensors-20-02719-s001.pdf]

sensors

Supplementary Materials

Towards the Development of a 3-D Biochip for the Detection of Hepatitis C Virus

Mariia Antipchik [1], Dmitry Polyakov [2], Ekaterina Sinitsyna [1], Apollinariia Dzhuzha [3], Mikhail Shavlovsky [2], Evgenia Korzhikova-Vlakh [1,3,*] and Tatiana Tennikova [3]

1. Institute of Macromolecular Compounds, Russian Academy of Sciences, Bolshoy pr. 31, 199004, St. Petersburg, Russia; volokitinamariya@yandex.ru (M.A.); kat_sinitsyna@mail.ru (E.S.)
2. Institute of Experimental Medicine, Academician Pavlov's str. 12, 197376, St. Petersburg, Russia; ravendoctor@mail.ru (D.P.); mmsch@rambler.ru (M.S.)
3. Institute of Chemistry, Saint-Petersburg State University, Universitesky pr. 26, Petrodvoretz, 198584, St. Petersburg, Russia; polinadzhuzha@mail.ru (A.D.), tennikova@mail.ru (T.T.)

* Correspondence: vlakh@mail.ru

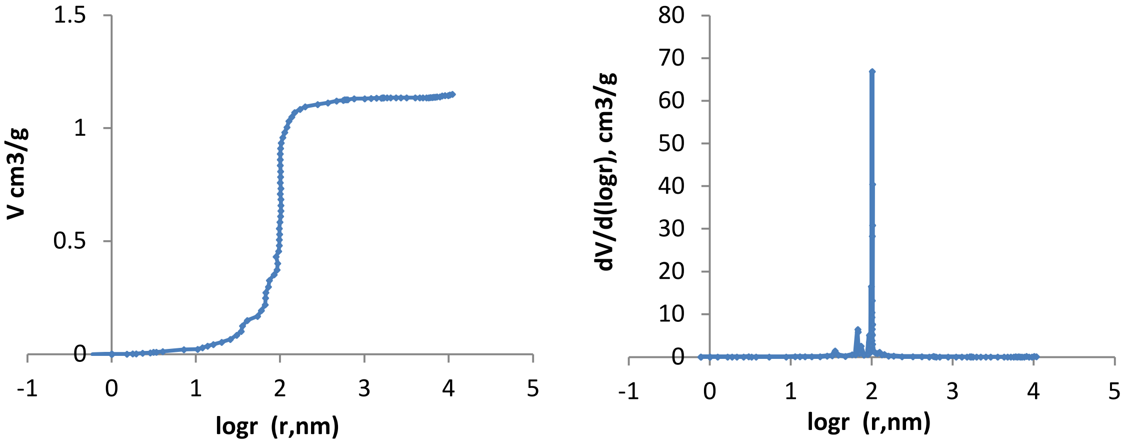

Characteristics	Value
Average pore radius (diameter), nm	183 (366)
Porosity over weight, cm³/g	1.2924
Porosity over volume, cm³/cm³	0.6071
(**A**)	(**B**)

Figure S1. Integral (**A**) and differential (**B**) distribution of pore volume (V) versus pore radius (log r).

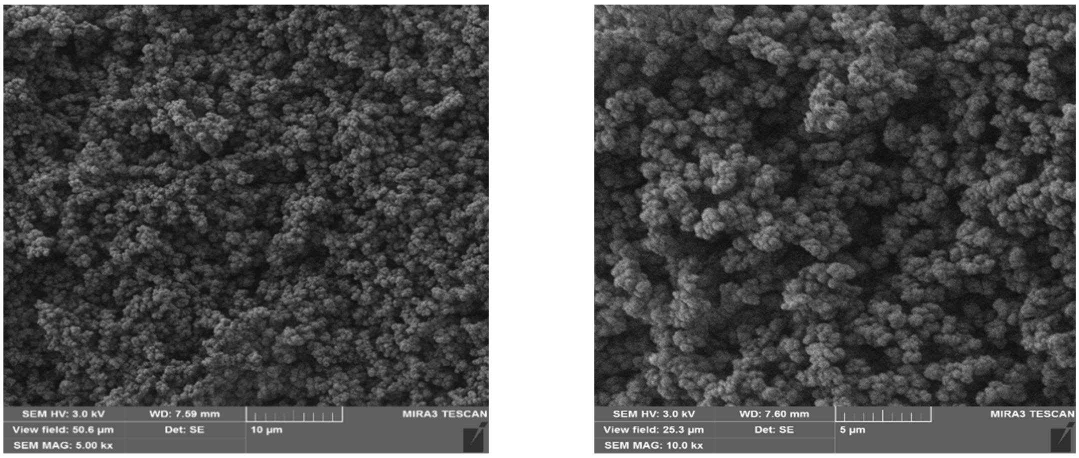

× 5000 **× 10,000**

Figure S2. SEM image of macroporous monolithic material based on P(GMA-*co*-DEGDMA) (carbon deposition).

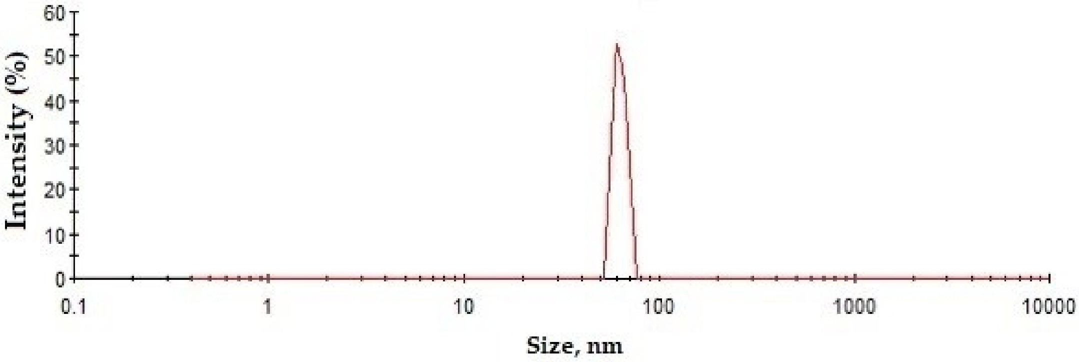

Figure S3. Dynamic light scattering of PLA-*b*-PEG nanoparticles used for VMPs′ preparation.

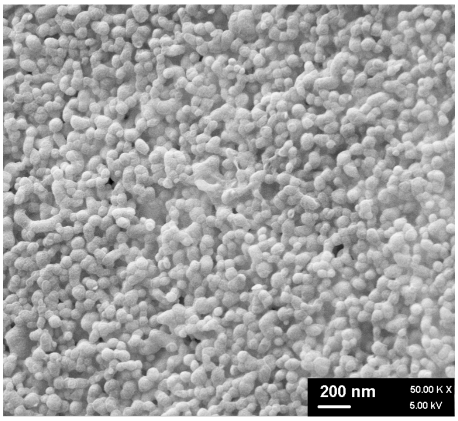

Figure S4. SEM image of PLA-*b*-PEG-based VMPs (gold deposition).

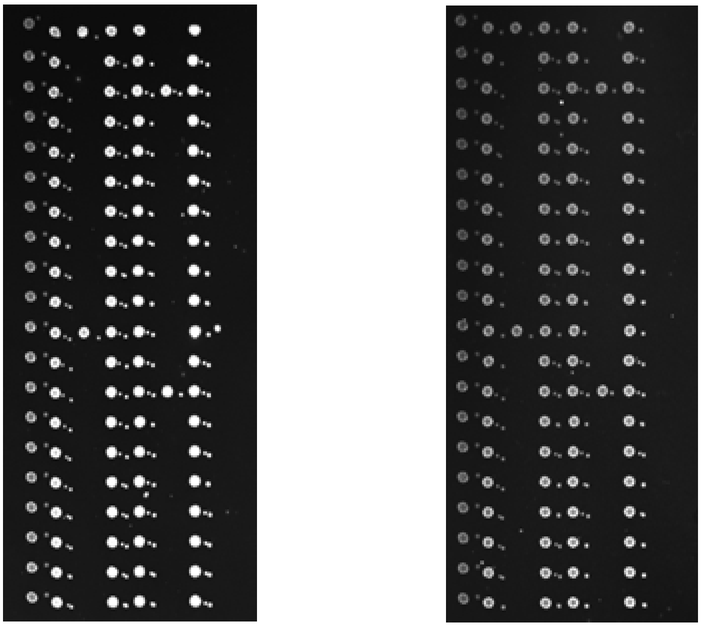

(A) **(B)**

Figure S5. Images of scanned biochips (fragments): (A) CD81-LEL (probe) – E2 (analyte); (B) CD81-LEL (probe) – VMPs (analyte). Spots with different concentration of analytes (the increase of concentration from left to right).
